# A 3D Printed Composite Scaffold Loaded with Clodronate to Regenerate Osteoporotic Bone: In Vitro Characterization

**DOI:** 10.3390/polym13010150

**Published:** 2021-01-01

**Authors:** Stefania Cometa, Maria Addolorata Bonifacio, Elisabetta Tranquillo, Antonio Gloria, Marco Domingos, Elvira De Giglio

**Affiliations:** 1 Jaber Innovation s.r.l., Via Calcutta 8, 00100 Roma, Italy; stefania.cometa@jaber.it; 2 Department of Chemistry, University of Bari Aldo Moro, Via E. Orabona 4, 70126 Bari, Italy; maria.bonifacio@uniba.it; 3 INSTM, National Consortium of Materials Science and Technology, Via G. Giusti 9, 50121 Florence, Italy; 4 Department of Mechanical, Aerospace and Civil Engineering & Henry Royce Institute, School of Engineering, Faculty of Science and Engineering, University of Manchester, Manchester M13 9PL, UK; elisabettatra90@gmail.com; 5 Institute of Polymers, Composites and Biomaterials, National Research Council of Italy, V.le J.F. Kennedy 54-Mostra d’Oltremare Pad. 20, 80125 Naples, Italy; antonio.gloria@cnr.it

**Keywords:** additive manufacturing, composite scaffold design, bone substitute, poly(ε-caprolactone), hydroxyapatite, clodronate, mechanical analysis, biocompatibility, thermal analysis, X-ray Photoelectron Spectroscopy

## Abstract

Additive manufacturing (AM) is changing our current approach to the clinical treatment of bone diseases, providing new opportunities to fabricate customized, complex 3D structures with bioactive materials. Among several AM techniques, the BioCell Printing is an advanced, integrated system for material manufacture, sterilization, direct cell seeding and growth, which allows for the production of high-resolution micro-architectures. This work proposes the use of the BioCell Printing to fabricate polymer-based scaffolds reinforced with ceramics and loaded with bisphosphonates for the treatment of osteoporotic bone fractures. In particular, biodegradable poly(ε-caprolactone) was blended with hydroxyapatite particles and clodronate, a bisphosphonate with known efficacy against several bone diseases. The scaffolds’ morphology was investigated by means of Scanning Electron Microscopy (SEM) and micro-Computed Tomography (micro-CT) while Energy Dispersive X-ray Spectroscopy (EDX) and X-ray Photoelectron Spectroscopy (XPS) revealed the scaffolds’ elemental composition. A thermal characterization of the composites was accomplished by Thermogravimetric analyses (TGA). The mechanical performance of printed scaffolds was investigated under static compression and compared against that of native human bone. The designed 3D scaffolds promoted the attachment and proliferation of human MSCs. In addition, the presence of clodronate supported cell differentiation, as demonstrated by the normalized alkaline phosphatase activity. The obtained results show that the BioCell Printing can easily be employed to generate 3D constructs with pre-defined internal/external shapes capable of acting as a temporary physical template for regeneration of cancellous bone tissues.

## 1. Introduction

The rise of Additive manufacturing (AM) technologies in regenerative medicine (RM) has greatly enhanced our ability to develop 3D constructs able to replicate the complex structural and functional architecture of native tissues and organs [1,2]. Operating in a layer-by-layer fashion, AM technologies are instrumental for the design of scaffolds with pre-set internal/external organization and fully interconnected networks of porous channels [3,4]. 3D printing belongs to the Biofabrication techniques and encompasses the processing of both cellular and acellular materials into biological constructs for RM, disease modelling or more conventional tissue engineering (TE) applications [5]. Over the last decade several bioprinting techniques including material extrusion, material jetting and vat photopolymerization have been proposed and employed with relative success in the medical field [6,7,8]. Whilst their full potential remains unexplored, bioprinting systems are already prompting a gradual shift in terms of the clinical landscape where the combination of AM with imaging technologies is expected to allow for the rapid production of patient-specific implants with superior regeneration performance [9]. To facilitate this process and accelerate the translation of products from the bench to the bed side our group has recently developed a multi-functional bio-fabrication platform called BioCell printing. Through a sequential and fully automated process, multi-material constructs can be printed, sterilized, seeded with cells and cultured under dynamic conditions prior to in vivo implantation [10,11]. The integration and synchronization of these multiple stages into a single device carries several benefits for the engineering process of tissue constructs including a reduced risk of contamination, higher throughput rate and higher reproducibility. For the purpose of this work only stages 1 and 2 (i.e., printing and sterilization) were employed. 

Poly(ε-caprolactone) (PCL) is an FDA-approved, semi-crystalline polymer widely exploited to fabricate scaffolds for bone regeneration, because of its biocompatibility, long-lasting mechanical features and degradation properties [12,13,14,15]. The in vivo degradation of PCL scaffolds is mainly driven by hydrolytic mechanisms, with a degradation rate that could be adjusted to fit the regrowth of bone tissue [16,17,18]. However, the implantation of polymeric bone substitutes is not a straightforward process, because poor adhesion to the surrounding tissues is often accompanied by the scaffold encapsulation into fibrous tissue and in situ inflammatory reactions [19]. Consequently, the PCL mechanical performance is often reduced during scaffold degradation [20]. However, the incorporation of hydroxyapatite (HA) particles into polymeric matrices can be considered as an effective strategy to improve both mechanical and osteoconductive properties of materials for bone replacement [21,22]. Simultaneously, HA works as a pH buffer neutralizing the acidic by-products of the polymeric matrices, thus reducing local inflammation [23]. Bisphosphonates (BPs) are a class of compounds commonly exploited to treat several metabolic bone diseases, characterized by bone resorption (e.g., osteoporosis, cancer or congenital diseases) [24,25]. BPs share a common P-C-P moiety, in which P is a phosphonate group. The latter is crucial to bind HA and for the drug’s mechanism of action [26,27]. Indeed, due to their chemical attraction with metal cations, BPs strongly bind HA calcium phosphate crystals, inhibiting their accretion [28,29,30].

BPs mechanism of action is based on their impact on bone mineral and cellular phases [31]. Indeed, BPs target osteoclasts resorptive activity, while promoting osteoblasts viability and metabolic activity [32,33]. As far as BPs effects on osteoclasts are concerned, the nitrogen-containing BPs interfere with the pathway of mevalonic acid [34,35]. Thus, osteoclasts migration, adhesion and bone resorptive activities are hindered, leading to apoptosis [36]. Even though BPs are commonly administered drugs to treat several systemic metabolic bone diseases, the oral and intravenous administration of BPs does not allow to obtain high bioavailability rates. The poor BPs bioavailability is caused by degradation of the drugs in the gastrointestinal tract, combined with the accelerated clearance from the damaged bone site [37]. In this scenario, a local treatment by drug delivery systems (DDS), i.e., microspheres and scaffolds, can be seen as a more viable alternative route to administer the required BPs concentration in situ [38]. However, BPs are extremely hydrophilic and, for this reason, are hard to distribute homogeneously as bare molecules in a polymeric matrix, resulting in a low encapsulation efficiency, unless a proper delivery system is employed [39]. To meet this need, Kolmas and colleagues synthetized a composite system, based on biodegradable polyurethanes and HA, to control clodronate (CD) in situ delivery [40]. Similarly, Chen et al. proposed a composite bone filler, consisting in PCL/HA microspheres, for alendronate local administration [41]. In a similar vein, Puppi and co-workers developed an electrospun mesh made of PCL, HA and CD [42]. The prepared fibrous scaffold was able to tune CD delivery, enhancing the material osteoconductivity. However, for large bone defects and osteoporotic diseases, the opportunity to adjust the mechanical features of the engineered scaffold to fit those of human bone tissue becomes of great importance. Differently from other more conventional manufacturing technologies, this can be easily achieved with 3D printing by precise tuning the pore size, geometry and overall porosity of the constructs. Therefore, this work proposes for the first time, the development of a 3D printed composite scaffold composed of PCL, HA and CD as a load bearing tissue substitute for osteoporotic patients. As shown in previous works, the mechanical properties of the scaffolds can be easily tuned changing process and design parameters, but more importantly, this new system can act simultaneously as a temporary load bearing substitute whilst delivering drugs locally to the targeted tissue (i.e., bone) [43,44].

In this preliminary study, 3D printed scaffolds have been characterized for their thermal properties by Thermo-Gravimetric Analysis (TGA). Their morphological properties have been investigated by micro-Computed Tomography (micro-CT) image analysis, Scanning Electron Microscopy (SEM) and, for elemental composition, by Energy Dispersive X-ray Spectroscopy (EDX). Moreover, in order to achieve a complete physicochemical understanding of the composite materials, proposed for osteoporotic bone regeneration, their surface composition has been further studied by X-ray Photoelectron Spectroscopy (XPS). Static compression tests were also carried out to evaluate the mechanical response of printed constructs and compared against those of native bone tissue. The scaffold potential to promote human mesenchymal stem cells attachment, spreading and differentiation was also evaluated.

## 2. Materials and Methods 

### 2.1. Materials

Granular poly (ε-caprolactone) (PCL, CAPA 6500) with molecular weight of 50,000 was purchased from Perstorp UK Ltd. (Cheshire, UK) and used without any further modification. Synthetic HA (nanoXIM HAp 402) with micrometric granulometry (5 ± 1 µm) was supplied by Fluidinova S.A. (Moreira da Maia, Portugal). CD, disodium salt in the form of powder, together with all the solvents and chemical reagents, were purchased from Sigma-Aldrich (Milan, Italy) and used as received.

### 2.2. Physical Blends Preparation

**Figure 1:**
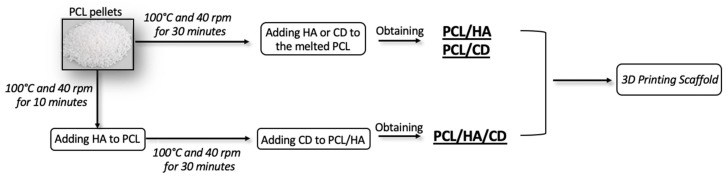
A flow diagram lustrating the mixing process for the generation of PCL, PCL/HA, PCL/CD and PCL/HA/CD blends.

The melt blending method was carried out using a Plastograph^®^ EC mixer (Brabender^®^ GmBH & Co. KG, Germany). Poly-ε-caprolactone (PCL) was initially molten at 100 °C and 40 rpm, for 10 min. Successively, the weighted amount of HA (20 wt. %) or CD (2 wt. %), previously dried in an oven at 100 °C for 2 h, were added to the molten PCL in the Brabender at 100 °C and 40 rpm for 30 min, obtaining a complete homogenization of the mixture PCL/HA and PCL/CD, respectively. PCL/HA/CD blends were obtained following the same procedure of PCL/HA blend but with the addition of CD (2 wt. %) being done at the end and followed by 30 min of homogenization at 100 °C and 40 rpm. Finally, PCL/HA, PCL/CD and PCL/HA/CD films were obtained using a hydraulic press. The films were then cut into small pellets and placed inside a vacuum desiccator for 24 h before being used for the printing of the different scaffolds (Figure 1). The PCL/HA/CD ratio reported in this study was selected on the basis of previous works performed by our group [45] and Puppi et al. [42,46,47].

### 2.3. Scaffold Fabrication

Scaffolds were fabricated using a 3D printing system developed in-house (i.e., BioCell Printing) as previously described elsewhere [10]. Using CAD software (SolidWorks, Dassault Systèmes, see Figure S1 in Supplementary Material), quadrangular prism withs 30 mm(length) × 30 mm(width) × 8 mm (height), were initially designed and subsequently sliced into multiple layers of homogenous thickness (.sli file). This information was then sent to the pattern-generator software where a single 0°/90° lay down pattern was imposed to create square-pore geometries. Using a nozzle with 300 μm of internal diameter and setting all process parameters as described in Table 1, 3D scaffolds were then printed layer-by-layer in a fully automated fashion and sterilized with UV light using the BioCell printing system. 

### 2.4. Scaffold’s Characterization

#### 2.4.1. Thermal Analysis

PCL-based scaffold’s thermal properties were evaluated by differential scanning calorimetry (DSC). The analysis was carried out using a Mettler DSC 822C module with FRS5 sensor (Mettler Toledo, Milan, Italy), using the STAR^e^ software (Mettler Toledo, Milan, Italy). Aliquots of about 10 mg were scanned from −100 °C to 100 °C, at a rate of 10 °C/min, including first and second heating and first cooling under nitrogen flux.

Peaks of the melting endotherms identified melting temperatures (Tm), while the glass transition temperatures (Tg) were calculated as the inflection point of the specific heat capacity. The enthalpies of fusion (ΔH_m_) were achieved calculating the areas under the melting peaks. Indium and silver specimens were used as calibration standards. The crystallinity degree, coded as X_c_ (%), was calculated using the following equation:

$$X_{c}\left(\%\right)\;=\;\left(\frac{{\Delta H}_{m}-{\;\Delta H}_{c}}{{\omega \Delta H}_{m}^{0}}\right)\;\times \;100$$

where ΔH_m_ represents the experimental melting enthalpy, ΔH_c_ is the cold crystallization enthalpy, ω is the weight fraction of material [48]. Furthermore, as reported by Woodruff et al. [49], ΔH^0^_m_, i.e., the melting enthalpy of 100% crystalline PCL, was assumed to be equal to 139 J/g. 

The thermal behaviour of the blends was studied employing a thermogravimetric analyser, PerkinElmer TGA-400 instrument (PerkinElmer Inc., Waltham, MA, USA). The temperature calibration was performed in the range of 30–600 °C, heating at 10 °C/min under nitrogen. At least three runs were carried out for each specimen, having weight in the range 5–10 mg.

#### 2.4.2. Micro-Computed Tomography (Micro-CT)

The SkyScan 1072 system (Aartselaar, Belgium) was used for computed tomography (micro-CT) analysis of 3D printed scaffolds, with a rotation pitch of 0.9° over a 180° angle. Various types of software, such as the abovementioned SkyScan software package, Image J software (NIH, Bethesda, MD, USA) and Mimics software (Materialise, Leuven, Belgium ) were used to reconstruct the cross-sections, 3D scaffold representations, to evaluate the porosity of the scaffold, surface area to volume ratio and the interconnectivity (Table 2).

#### 2.4.3. Scanning Electron Microscopy (SEM) with Energy Dispersive X-ray Spectroscopy (EDX)

JEOL microscope (JSM5600LV, Tokyo, Japan) was used to observe the scaffolds fractured surfaces, obtained in liquid nitrogen. The coating with Au on an SEM coating device (Edward Sputter Coater) was applied before the observation. The sample surface was coated with a homogeneous layer of metal of 5–6 nm thickness.

The scaffolds not metalized were used for the microanalysis with an element mapping distribution by the energy dispersive X-ray microanalysis spectroscopy accessory (EDX-Oxford Instrument). Captured images were analysed by FiJi software (ImageJ, NIH, Bethesda, MD, USA) to measure PCL filament thickness, pore sizes and particles distribution into the composite scaffolds.

#### 2.4.4. X-ray Photoelectron Spectroscopy (XPS)

X-ray photoelectron spectroscopy (XPS) was carried out to study the surface composition of PCL-based materials. Spectra were obtained using a scanning microprobe PHI 5000 VersaProbe II equipped with a monochromatized AlKα X-ray radiation source (Physical Electronics, MN, USA). The base pressure of the instrument was 10^−9^ mbar. Spectra were recorded in HP mode (sampled area ~1400 × 200 µm^2^), with an X-ray take-off angle of 45°. For each analysis, survey scans and high-resolution spectra were recorded in FAT mode (pass energy 117.4 eV and 29.35 eV, respectively). 

Data analysis was performed with MultiPak software (v. 9.9.0, ULVAC-PHI Inc, Chanhassen, MN, USA), a non-linear least-square fitting program. Gaussian-Lorentzian peaks having the same full width at half maximum (FWHM) were set. The lower binding energy of C1s photo-peak (e.g., C1s hydrocarbon peak) was fixed as charge reference at 284.8 eV. The Normalized peak areas were exploited to calculate atomic percentages (At%). Furthermore, empirically derived sensitivity factors, in accordance with MultiPak library, were used to normalize peak areas and, to compare data belonging to different elements.

#### 2.4.5. Mechanical Analysis

Compression tests were carried out on 3D printed scaffolds using block-shaped specimens (length—L_0_ of 5.0 mm, width—W_0_ of 5.0 mm, height—H_0_ of 8.0 mm). The specimens were compressed to a strain of 50% at a rate of 1 mm/min, using an INSTRON 5566 testing machine. Taking into account the measured force F and the initial cross-sectional area of the specimen (A_0_ = L_o_ · W_0_), the ‘‘apparent’’ stress (σ) was calculated as follows:

$$\sigma \;=\;\frac{F}{A_{0}}$$

The strain (ε) was evaluated as the ratio between the height variation (ΔH) of the specimen and the initial height (H_0_):

$$\varepsilon \;=\;\frac{\Delta H}{H_{0}}$$

#### 2.4.6. Biological Analysis

##### Cell Culture

Human mesenchymal stem cells (hMSCs, Millipore, Germany), at the fourth passage, were cultured in DMEM (Microtech, Italy) supplemented with 10% (*v*/*v*) FBS (Gibco™, Thermo Fisher Scientific), 2 mM L-glutamine and antibiotics (penicillin G sodium 100 U/mL, streptomycin 100 g/mL) at 37 °C and 5% CO₂. PCL, PCL/CD, PCL/HA and PCL/HA/CD scaffolds were sterilized by soaking the structures in a solution of ethanol and antibiotics (penicillin/streptomycin), washed in PBS (Sigma–Aldrich, Milan, Italy) and pre-wetted in FBS. hMSCs were seeded onto the scaffolds and a density of 1.0 × 10⁴ cells/sample was employed. The cell-laden scaffolds were incubated for 2 h (37 °C, 5% CO₂) and culture medium was then added to each well in a multi-well plate.

##### Alamar Blue Assay

The Alamar Blue assay (AbD Serotec Ltd., Kidlington, UK) was employed to analyse cell viability and proliferation. At 1, 3 and 7 days after cell seeding, the different kinds of cell-laden scaffolds were rinsed with PBS (Sigma–Aldrich, Milan, Italy), and DMEM without Phenol Red (HyClone, Cramlington, UK) containing 10% (*v*/*v*) Alamar Blue was added for each sample. The cell-laden scaffolds were incubated for 4 h (37 °C, 5% CO_2_). 

A spectrophotometer (Sunrise, Tecan, Männedorf, Zurich, Switzerland) was used to measure the optical density at specific wavelengths (570 and 595 nm). The percentage of Alamar Blue reduction was evaluated at different time points. The experiments were done at least three times in triplicate.

##### Alkaline Phosphatase Activity 

Samples were removed from the medium and washed twice with PBS at 7, 14 and 21 days. The cell-laden scaffolds were then incubated in lysis buffer and centrifugated. The SensoLyte pNPP alkaline phosphatase assay kit (AnaSpec Inc., Fremont, CA, USA) was used to measure the alkaline phosphatase (ALP) activity. The Quant-iT PicoGreen assay kit (Molecular Probes Inc., Eugene, OR, USA) was also employed for DNA detection and quantification. The normalized ALP activity (ALP/DNA) was then evaluated. All the solutions were prepared according to the manufacturer’s protocol and the procedure was properly followed. The experiments were performed at least three times in triplicate.

#### 2.4.7. Statistical Analysis

The experimental data were reported as mean value ± standard deviation. Statistical analysis was made by ANOVA followed by Bonferroni post hoc test. Statistical significance was set at *p* < 0.05.

## 3. Results and Discussion

### 3.1. Thermal Characterization

DSC analysis was performed on PCL-based blends in order to ascertain changes in thermodynamic properties of PCL due to the addition of the microsized inorganic filler. The values of Tg, Tm, enthalpy and crystallinity level, for processed PCL and PCL/HA systems are presented in Table 3. It was observed that the glass transition and fusion temperatures relevant to PCL/HA composite scaffolds resulted similar to those relevant to pure PCL scaffold. On the other hand, in PCL/HA blends, the crystallinity level was lower than the crystallinity of PCL, as expected due to the presence of a significant amount of HA. Results relevant to PCL/HA-CD resulted quite similar to PCL/HA, and therefore were not reported.

**Figure 2:**
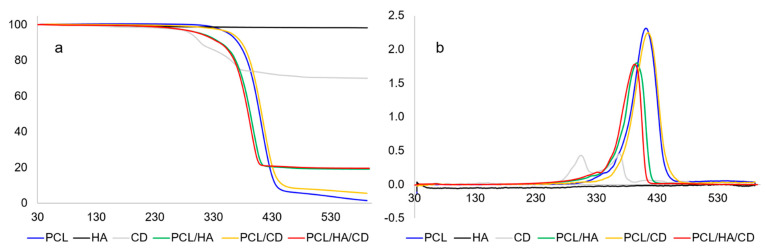
Thermogravimetric (**a**) and derivative (**b**) curves of pure materials and scaffolds.

Moreover, the thermal stability of the blends was analysed by thermogravimetry (TGA). TGA was employed to evaluate possible changes in material properties due to the incorporation of the inorganic compound and/or the drug into the polymer matrix. The thermogravimetric and derivative curves of both the feed materials and fabricated scaffolds are shown in Figure 2, while the main quantitative results related to these measurements are reported in Table 4. 

First, it was evaluated whether the PCL underwent alterations owing to the extrusion process. Indeed, for fabrication of these composite scaffolds, PCL was exposed to two heating processes, i.e., the melt mixing with the other components of the composite and the heat extrusion process. Comparing the thermogram of PCL scaffold to that of pure PCL (data not reported), no differences were detected. As far as micrometric HA thermal behaviour is concerned, no significant weight losses due to temperature were observed, as evidenced by the high residue at 600 °C. On the other hand, the CD molecule evidenced two thermal events, the first at 304 °C and the second at 368 °C, with an important residue at 600 °C.

Taking into account the PCL-based scaffolds, it is noteworthy to consider that the addition of CD did not dramatically change the degradation temperature of PCL, while the presence of micrometric HA decreased the thermal stability of the scaffold. Indeed, the peak temperatures of the PCL composite scaffolds containing HA (i.e., PCL/HA and PCL/HA/CD) resulted lower than the peak temperature of the pure PCL scaffold, as already reported in literature [50]. The PCL degradation mechanisms occur in two phases. In the first phase, the breaking of the polyester chains is involved through the pyrolysis of the ester bond, while in the second phase the formation of ε-caprolactone is attributed by means of a decompressed depolymerization process [51]. Probably, the interaction between hydroxyapatite and the functional groups of the PCL, causes a weakening of the bonds within the PCL chain, which leads to an advance of polymer degradation reactions. However, it is important to note that all processing temperatures used for the extrusion of PCL-based blends are well below the onset temperatures of the scaffolds (in any case, higher than 200 °C), thus ensuring the printing of 3D scaffolds without deterioration of the polymeric matrix. Another important feature is the estimation of the PCL/HA and PCL/HA/CD residues at 600 °C, which are mostly indicative of the inorganic matter. The observed values, reported in Table 4, seem to agree with their feed ratios in the mixture (i.e., 20% and 19.6% for PCL/HA and PCL/HA/CD, respectively). This means that the melt blending process did not result in any HA mass loss. 

### 3.2. Morphological Characterization 

#### 3.2.1. Micro-Computed Tomography (Micro-CT)

**Figure 3:**
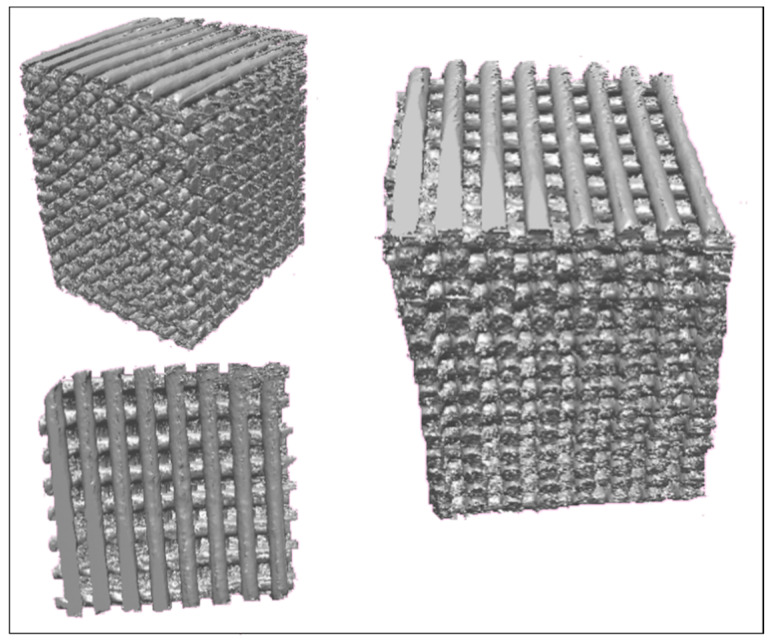
3D reconstructions obtained from micro-CT analysis on PCL scaffolds with a 0/90° lay down pattern.

All scaffolds, regardless of the content of HA and CD, showed a well-organized structure with a definite size and shape of pores and a repeatable microstructure, as confirmed by micro-CT (Figure 3). The micro-CT analysis showed that the real and theoretical values related to the diameter of the filament and the size of the pores have sufficient coherence. In fact, the average fibre diameter, despite being slightly above the internal diameter of the printing nozzle (i.e., 300 µm), is constant and of 350 µm. The imposed centre-to-centre distance of 750 µm between two filaments in the same layer, resulted in scaffolds with consistent pore sizes of approx. 350 µm and porosity level of 58%. Importantly, all the scaffolds have clearly shown a totally interconnected pore network (scaffold interconnectivity: 100%). The presence of HA and/or CD did not affect the porosity and interconnetivity values observed for pure PCL scaffold.

#### 3.2.2. Scanning Electron Microscopy (SEM)

**Figure 4:**
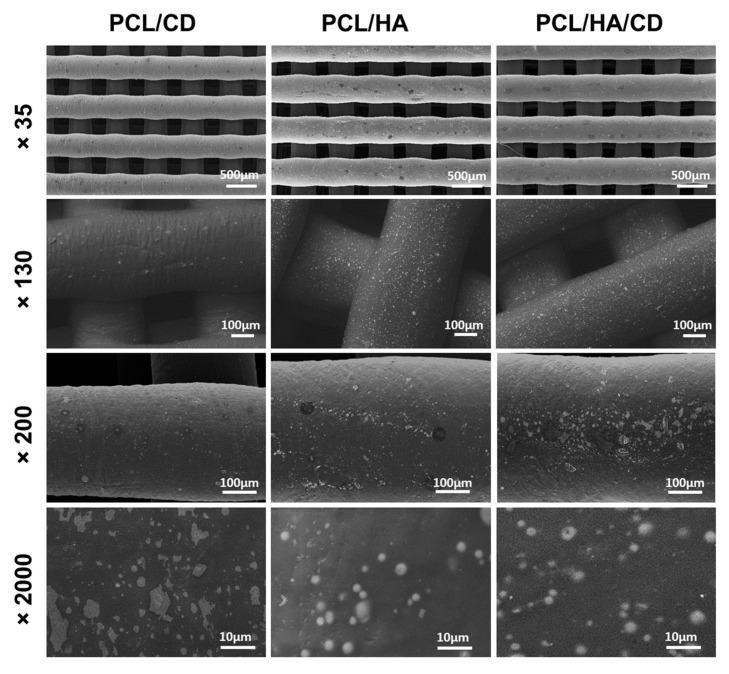
SEM micrographs at different magnifications (×35, ×130, ×200 and ×2000) of composite PCL/CD (left column), PCL/HA (middle column), and PCL/HA/CD (right column) scaffolds.

SEM micrographs, shown in Figure 4, revealed a homogeneous distribution of HA and CD inside the composite PCL scaffolds, successfully fabricated by *BioCell* Printing. All the scaffolds displayed highly ordered, square-shaped pores with dimensions in the range of 350–360 µm, as previously defined during the design stage. This hierarchical, microporous architecture, according to the literature, is suitable for cell colonization, tissue ingrowth and vascularization [52,53,54]. The presence of HA and CD did not significantly alter the porosity of the composite, as well as the diameter of the printed PCL filaments, which remained constant (approx. 350 µm) and in agreement with the diameter of the nozzle used for extrusion. HA powder formed aggregates of porous microparticles (approx. 6.5 µm^2^), well dispersed onto the PCL filaments. Moreover, the SEM image of PCL/HA/CD composite showed aggregates with irregular sizes (from 0.2 to 2.8 µm^2^) and generally smaller than those found in PCL/HA scaffold. Conversely, the bare drug in PCL/CD composite formed randomly distributed islet-like structures of different sizes, ranging from 1.9 to 14.5 µm^2^.

Moreover, EDX analyses highlighted the presence of HA or CD particles within the filaments. Indeed, EDX analysis reported in Table 5 revealed an elemental composition of the PCL/HA blends with a Ca/P ratio equal to 1.8. On the other hand, in PCL/HA/CD sample, a Ca/(P-Cl) equal to 1.6 was obtained (considering that in a CD molecule the P/Cl ratio is 1:1, subtracting to the total P% that of Cl, we hypothetically obtained the atomic % of P relevant to HA). Considering the experimental error, a Ca/P ratio in the range of 1.6-1.8 can be considered a good approximation of the typical Ca/P ratio of hydroxyapatite molecules.

### 3.3. XPS Analysis

The surface characterization of the PCL-based scaffolds was performed by means of XPS, in order to assess the surface elemental composition of the blends and the surface availability of the bisphosphonate added to the mixture. In Table 6, the surface composition, expressed as atomic percentage, of PCL/CD, PCL/HA and PCL/HA/CD scaffolds was reported.

The Ca2p/P2p corrected area ratio, obtained by XPS analysis, was 1.66 ± 0.08 for the PCL/HA blend. For PCL/CD sample, a P2p/Cl2p/Na1s corrected signal area ratio of 1.0:1.1:1.1 was obtained (versus a theoretical P/Cl/Na ratio of 1:1:1), thus confirming the protection of the drug during the scaffold production. Additionally, the pure drug presented a similar signal area ratio (i.e., 1.0:0.9:1.0). As far as the PCL/HA/CD scaffold surface composition is concerned, a Cl2p/Na1s ratio of 1.1 was obtained, suggesting the prevention of CD degradation also in the blend containing HA. Considering that in PCL/HA/CD the total P2p corrected signal area was relevant both to HA and CD moieties, and assuming that the P2p corrected signal area relevant only to CD could be considered equal to that of Cl2p (or Na1s), the P2p due to HA can be obtained by the following equation:

$$P_{2p}^{HA}\;=\;P_{2p}^{tot}-P_{2p}^{CD}\;=\;P_{2p}^{tot}-\;Cl_{2p}\;$$

**Figure 5:**
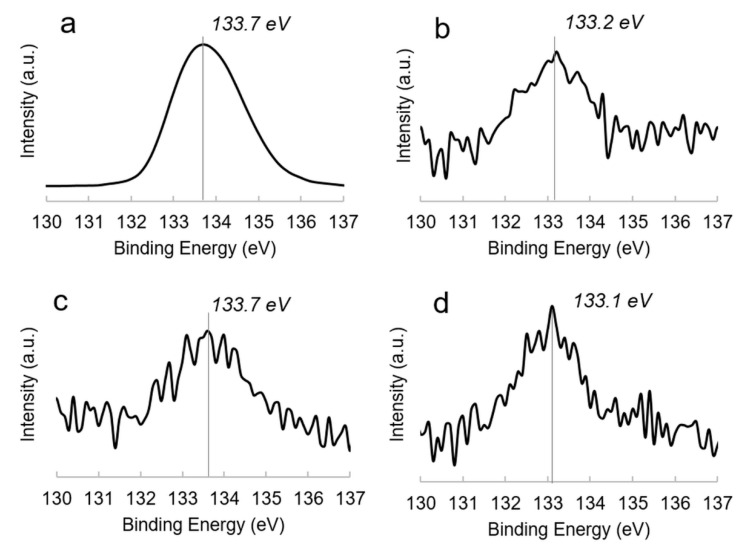
P2p high-resolution spectra of CD (**a**), PCL/HA (**b**), PCL/CD (**c**) and PCL/HA-CD (**d**) samples.

Based on this calculation, the Ca2p/P2p^HA^ was equal to 1.72 ± 0.06. Therefore, from XPS analysis, as well as from EDX, we can conclude that a Ca/P ratio in the range 1.6–1.7 is in agreement with the stoichiometric ratio of HA (1.67). Another feature that should be highlighted was relevant to the P2p binding energies in PCL/HA, PCL/CD and PCL/HA/CD scaffolds (data collected over at least three replicates for each typology), whose high resolution spectra are reported in Figure 5. In PCL/HA (Figure 5b), P2p signal fell at 133.2 ± 0.2 eV, which was typical of hydroxyapatite, where phosphate groups are linked to calcium [55]. In PCL/CD (Figure 5c), the P2p signal fell at slightly higher binding energies (i.e., 133.7 ± 0.2 eV), as already found for other bisphosphonates [56] and detected on the pure drug (Figure 5a). When HA and CD were blended in the PCL/HA/CD system (Figure 5d), the P2p BE was found at 133.1 ± 0.2 eV, indicating that almost all the phosphate present on the surface, even if belonging to CD, was hydroxyapatite-like. This finding evidenced that the bisphosphonate could be present in the blend linked to the HA via chelation of its phosphate groups with the HA calcium ions.

### 3.4. Mechanical Analysis

**Figure 6:**
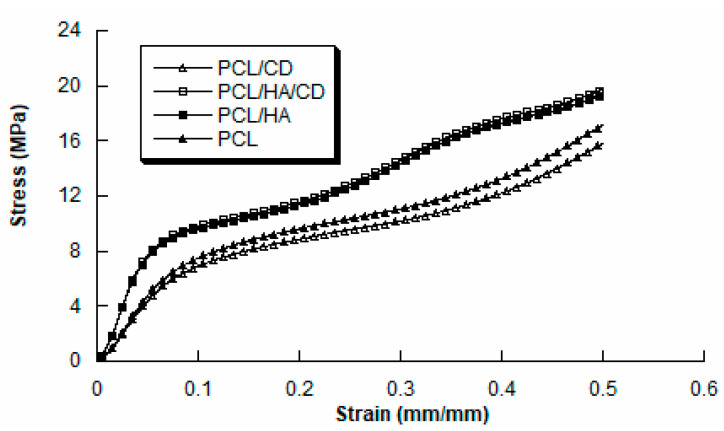
Representative stress-strain curves illustrating the mechanical response of PCL, PCL/CD, PCL/HA and PCL/HA/CD scaffolds (rate of 1 mm/min, final strain of 50%).

The mechanical analysis conducted on the different kinds of scaffolds showed a behaviour similar to that of a flexible foam [43]. Typical stress-strain curves are reported in Figure 6. The compressive modulus (E) was calculated as the slope of the initial linear region of the curve. Table 7 reports compressive modulus and maximum stress as mean value ± standard deviation. It was possible to observe that the compressive modulus and maximum stress increased from 106 ± 11 MPa to 220 ± 30 MPa and from 16.5 ± 1.4 MPa to 18.4 ± 1.2 MPa, respectively, when the HA was added in PCL. Moreover, PCL/HA/CD scaffolds showed values of compressive modulus (228 ± 27 MPa) and maximum stress (18.9 ± 1.3 MPa) which were significantly higher (*p* < 0.05) than those found for PCL/CD (108 ± 10 MPa and 15.9 ± 1.6 MPa). However, in terms of compressive modulus and maximum stress, no statistically significant differences (*p* > 0.05) were observed between PCL and PCL/CD, as well as between PCL/HA and PCL/HA/CD. Thus, the addition of CD to both PCL and PCL/HA matrices did not induce any significant change on the mechanical behaviour of the constructs. This was probably related to the very low concentration of CD (2 wt. %) compared to HA (20 wt. %) used in the preparation of PCL/CD and PCL/HA/CD, respectively.

The mechanical properties of bone are crucial to the role of the skeletal system in supporting movement while providing protection to vital organs. In this regard, the incorporation of HA into the PCL scaffold should mimic the mechanical behaviour of the bone tissue. In literature, it was reported that human cancellous bone shows a compressive strength of 4–12 MPa and a Young’s modulus of 0.02–0.5 GPa, which are lower than those found for cortical bone (100–230 MPa and 7–30 GPa) [57]. 

Our results suggest that PCL/HA/CD scaffolds produced via *BioCell* Printing displayed adequate mechanical properties for the regeneration of cancellous bone tissue, while further optimization will be required to achieve values of compressive modulus and strength similar to those found for cortical bone. The obtained values were consistent with those already reported for PCL (105.5 ± 11.2 MPa, 16.5 ± 1.4 MPa) and PCL/micro-HA (217.2 ± 21.8 MPa, 17.4 ± 1.8 MPa) in a previous work [45].

### 3.5. Biological Analysis

**Figure 7:**
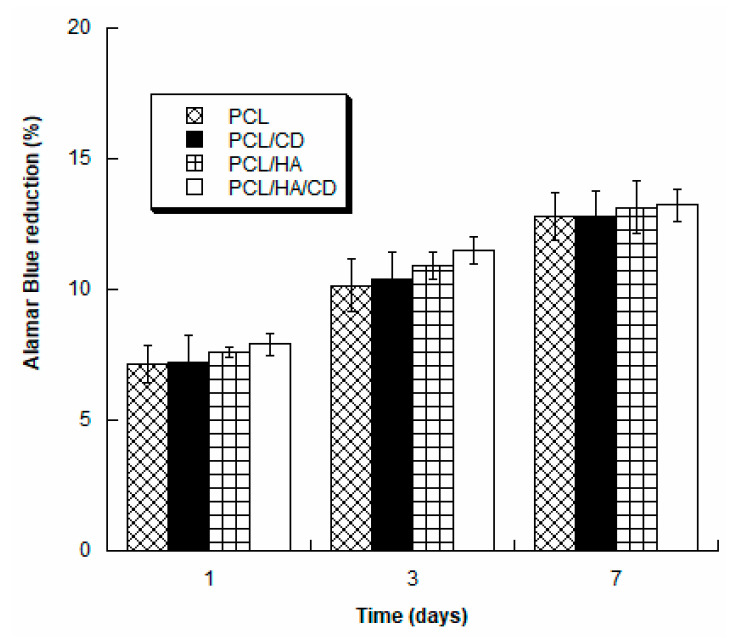
Alamar Blue reduction (%) evaluated for PCL, PCL/CD, PCL/HA and PCL/HA/CD at different time points.

Cell viability and proliferation were assessed for the different groups of 3D scaffolds (PCL, PCL/CD, PCL/HA, PCL/HA/CD). Figure 7 reports the obtained results as a percentage of Alamar Blue reduction.

For each group of cell constructs, the percentage of Alamar Blue reduction significantly increased over the analysed time period (*p* < 0.05). In general, the magnitude of dye reduction provides information on the number of viable cells and the findings indicated that all scaffolds supported the adhesion and proliferation of hMSCs. In addition, at each time point no statistically significant differences (*p* > 0.05) were observed among PCL, PCL/CD, PCL/HA and PCL/HA/CD, suggesting that the prepared scaffolds did not induce in vitro cytotoxicity. 

**Figure 8:**
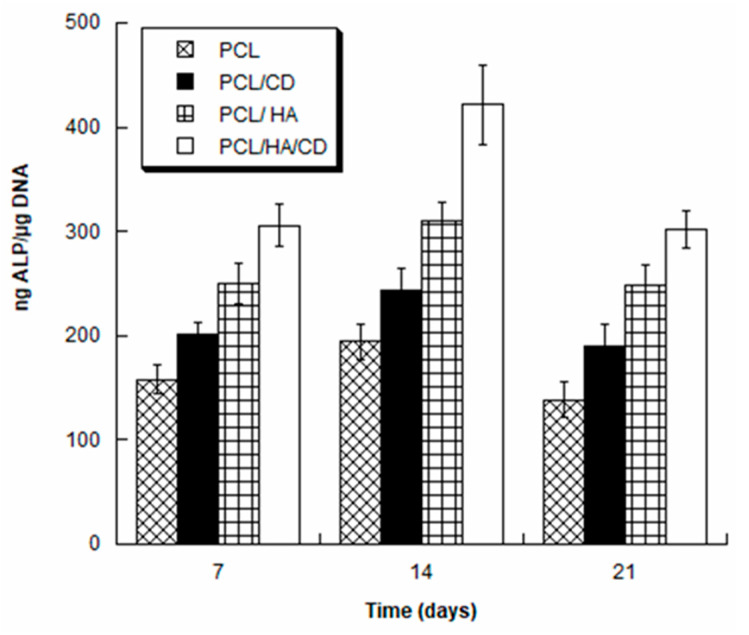
Normalized ALP activity (ALP/DNA) for PCL, PCL/CD, PCL/HA, PCL/HA/CD at 7, 14 and 21 days.

Furthermore, the normalized ALP activity (ALP/DNA) was measured at different time points (7, 14 and 21 days) for each group (PCL, PCL/CD, PCL/HA, PCL/HA/CD) in order to assess early osteogenic differentiation. The ALP/DNA ratio showed a peak value at 14 days for all groups (Figure 8).

In particular, at each time point, PCL showed lower values of normalized ALP activity if compared to PCL/CD, PCL/HA and PCL/HA/CD. Moreover, even though PCL/HA showed higher values in comparison to PCL/CD, they were lower than those found for PCL/HA/CD. The highest ALP values were observed for PCL/HA/CD at each tested time, demonstrating that HA and CD combination successfully supported osteogenic differentiation of hMSCs. The observed differences were statistically significant (*p* < 0.05). Future in vitro and in vivo studies will shed light on the composite scaffolds impact on osteoclasts-mediated bone resorption, providing further insights into the effectiveness of these 3D-printed systems.

## 4. Conclusions

The present study focuses on the fabrication and characterization of a composite scaffold based on PCL, loaded with HA and CD (PCL/HA(CD). The 3D printing and initial sterilization process of this scaffold have been performed, for the first time, by means of the *BioCell* Printing system, which demonstrated a high-precision reproduction of the model design. The obtained scaffold displayed proper microporosity and pore interconnectivity suitable for cell colonization, as highlighted by SEM-EDX and micro-CT analyses. Furthermore, HA microparticles were homogeneously distributed within the scaffold and as shown by thermal analyses, no mass loss was detected after the melt blending process. XPS data provided insights into the interactions between HA and CD. The latter seemed to be chelated by HA calcium cations, opening the opportunity to tune the in vivo drug release with a bone-HA displacement mechanism. The presence of CD did not negatively affect the mechanical performance of PCL/HA scaffolds nor the cell metabolic activity regarding the test time of seven days. Furthermore, in combination with HA, it determined an increase of the normalized alkaline phosphatase activity, thus indicating a pro-differentiating effect on hMSCs. Overall, the presented results highlight the opportunity to further investigate this composite scaffold for cancellous bone regeneration, also benefiting from the obtained mechanical and biological findings. 

## Figures and Tables

**Table 1:** Process/instrument parameters used to print PCL, PCL/HA, PCL/CD and PCL/HA/CD scaffolds. Deposition velocity (DV), slice thickness (ST), liquefier temperature (LT), extrusion pressure (EP), screw rotation velocity (SRV), (FD) filament distance and (FG) filament gap.

	DV (mm/s)	ST (mm)	LT (°C)	EP (bar)	SRV (r/min)	FD µm	FG µm
**PCL**	20	0.270	90	5	35	750	350
**PCL/HA**	20	0.270	100	5	28	750	350
**PCL/CD**	20	0.270	90	5	35	750	350
**PCL/HA/CD**	20	0.270	100	5	28	750	350

**Table 2:** Morphological properties of 3D printed scaffolds and formulae used for the evaluation of porosity, surface area to volume ratio, and interconnectivity [6].

Characteristics	Formulae/Definition
Porosity	100% × volume of pores/sum of volume of pores and scaffold material
Surface area to volume ratio	Surface area of scaffold struts volume of scaffold material
Interconnectivity	100% × volume of interconnected pores volume sum of interconnected and closed pores

**Table 3:** Thermodynamics characteristics of PCL during second heating scan.

Sample	Tg (°C)	Tm (°C)	ΔHm (J/g)	Xc%
PCL	−63.5	57.0	76.9	55.2
PCL/HA	−63.2	58.9	67.1	48.1

**Table 4:** Residue percentages and peak degradation temperatures obtained by TGA.

Sample	Residue at T = 600 °C (%)	T_peak_ (°C)
PCL	1.5 ± 0.4	410.4 ± 1.6
HA	98.2 ± 1.3	-
CD	57.7 ± 0.9	1st: 304 ± 2; 2nd: 368 ± 2
PCL/HA	19.6 ± 0.3	395.0 ± 1.4
PCL/CD	5.2 ± 1.9	414 ± 3
PCL/HA/CD	18.5 ± 0.8	392.9 ± 0.9

**Table 5:** Atomic percentages of PCL blends obtained by EDX analysis.

Element	Atomic %		
	PCL/CD	PCL/HA	PCL/HA/CD
C K	61.1	71.3	52.4
O K	25.3	19.6	38.6
P K	4.9	3.2	3.6
Ca K	-	5.9	5.4
Cl K	4.5	-	0.1
Na K	4.2	-	0.1

**Table 6:** Atomic percentages of PCL blends obtained by XPS analysis.

Element	Atomic %		
	PCL/CD	PCL/HA	PCL/HA/CD
C1s	76.1 ± 1.9	75.3 ± 0.2	75.2 ± 0.2
O1s	21.3 ± 1.9	23.1 ± 0.5	23.0 ± 1.7
P2p	0.15 ± 0.02	0.35 ± 0.02	0.31 ± 0.04
Ca2p	-	0.58 ± 0.07	0.31 ± 0.11
Si2p	2.2 ± 0.1	0.7 ± 0.6	0.9 ± 0.7
Cl2p	0.14 ± 0.02	-	0.13 ± 0.06
Na1s	0.13 ± 0.02	-	0.13 ± 0.07

**Table 7:** Mechanical properties of PCL, PCL/CD, PCL/HA and PCL/HA/CD scaffolds: compressive modulus (E) and maximum stress (σ_max_).

Scaffold	E (MPa)	σ_max_ (MPa)
PCL	106 ± 11	16.5 ± 1.4
PCL/CD	108 ± 10	15.9 ± 1.6
PCL/HA	220 ± 30	18.4 ± 1.2
PCL/HA/CD	228 ± 27	18.9 ± 1.3

## Data Availability

The data presented in this study are available within the current manuscript and the supplementary material.

## Supplementary Materials

The following are available online at https://www.mdpi.com/2073-4360/13/1/150/s1, Figure S1: a) Schematic representation of a cross section viewed in the XZ plane of the building process (RW: road width or filament diameter; FG: filament gap; ST: slice thickness; LG: layer gap; FD: filament distance); b) 3D illustration of the additive manufactured scaffolds characterized by a 0◦/90◦ lay-down pattern.
