# Supplementary material for: A 3D Printed Composite Scaffold Loaded with Clodronate to Regenerate Osteoporotic Bone: In Vitro Characterization
Source: Polymers (Basel). 2021 Jan 1;13(1):150. doi: 10.3390/polym13010150 (PMC7795460; doi:10.3390/polym13010150)
Supplement: Supplementary file 1 [file polymers-13-00150-s001.pdf]

Supplementary Material

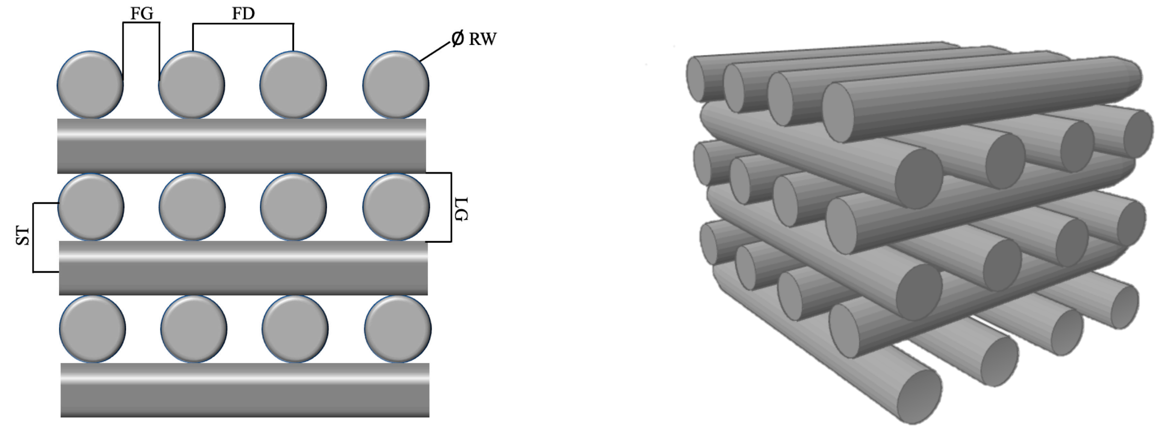

a) b)

Figure S1. (**a**) Schematic representation of a cross section viewed in the XZ plane of the building process (RW: road width or filament diameter; FG: filament gap; ST: slice thickness; LG: layer gap; FD: filament distance); (**b**) 3D illustration of the additive manufactured scaffolds characterized by a 0◦/90◦ lay-down pattern.
